# Common Genotypes of Hepatitis B virus prevalent in Injecting drug abusers (addicts) of North West Frontier Province of Pakistan

**DOI:** 10.1186/1743-422X-4-63

**Published:** 2007-06-28

**Authors:** Muhammad Masroor Alam, Sohail Zahoor Zaidi, Shehzad Shaukat, Salmaan Sharif, Mehar Angez, Asif Naeem, Shamim Saleha, Javed Aslam Butt, Salman Akbar Malik

**Affiliations:** 1 Department of Virology, National Institute of Health, Islamabad, Pakistan; 2 Head of Department of Virology; Principal Investigator-WHO Regional Reference Laboratory for Polio Eradication Initiative, National Institute of Health, Islamabad, Pakistan; 3 Research Student, Department of Virology, National Institute of Health, Islamabad Pakistan; 4 Head of Department of Gastroenterology, Pakistan Institute of Medical Sciences, Islamabad, Pakistan; 5 Head of Department of Biochemistry, Quaid-i-azam University, Islamabad, Pakistan

## Abstract

**Background:**

The epidemiological significance of Hepatitis B virus genotypes has been well established and becoming an essential concern day by day however, much little is known about the mixed infection with more than one Hepatitis B virus genotypes and their clinical relevance.

**Methods:**

Intravenous drug abusers are considered as a major risk group for the acquisition and transmission of blood borne infections like hepatitis B, however, in Pakistan, no such data has ever been reported about the epidemiology of HBV and its genotypes in Injecting Drug Users. 250 individuals were analyzed for hepatitis B virus genotypes after prior screening with serological assay for the detection of HBsAg.

**Results:**

56 (22.4%) individuals were found positive on ELSIA for HBsAg. The genotype distribution was found to be as: genotype D, 62.5%; genotype A, 8.92% while 28.57% individuals were found to be infected with a mixture of genotype A and D.

**Conclusion:**

There is an urgent need of the time to develop public health care policies with special emphasis towards the control of HBV transmission through high risk groups especially Injecting Drug Users.

## Background

Hepatitis B virus (HBV) infection is a well recognized and major health problem leading to significant morbidity and mortality worldwide especially in the developing countries. Approximately, 2 billion people in the world have been infected by HBV [1], 400 million of who are chronic carriers [2]. The virus causes acute hepatitis of varying severity [3] and persists in 95% of children and 2–10 % of adult patients [4] leading to chronic liver disease, cirrhosis, hepatocellular carcinoma [5] and even fulminant hepatitis [6]. In Pakistan, HBV infection rate is increasing day by day. The reason may be the lack of proper health facilities or poor economical status and less public awareness about the transmission of major communicable diseases like Hepatitis B virus, Hepatitis C virus and Human Immunodeficiency Virus.

The seropositivity rate of Hepatitis B surface antigen (HBsAg) varies in the different regions of the world with a considerable low rate in the developed countries like 0.6% in Wales, England, 1.2 % in USA. However, the developing countries depicts a high alarming rate reporting 19.6% in Egypt, 2–10% in India, 3.5% in Palestine and 1.6–7.7% in Brazil [7-11]. Viral hepatitis is endemic in Pakistan with an estimated rate of 3–4% [12]. Multiple studies have been conducted regarding prevalence rate of HBV infection based on various population groups in Pakistan. All such studies present a varying rate of infection based on the study design, population selected, diagnostic assays and demographical and epidemiological variation. According to various study groups, the HBV prevalence rate has been reported as 2–10% among healthy blood donors; 5–9% among health care personnel; 3.6–18.66% among the general population; 3.16% among the pregnant women; 10–20% in patients with provisional diagnosis of hepatitis and 3.16–10.4% among professional blood donors [13]. These reports highlight the lack of a country wide epidemiological studies that can present the overall disease status in the whole country.

Hepatitis B virus is a genetically heterogenous hepadnavirus possessing a partially double stranded DNA genome with an estimated rate of 1.4 – 3.2 × 10 ^-5 ^nucleotide substitution per site per year [14]. Additionally, the virus replicates by using its RNA polymerase which lacks the proof reading activity resulting in nucleotide misincorporations and an established genetic variability which gives rise to the well recognized subtypes and genotypes of the virus. Based on an inter-group divergence of 8% or more in the complete genomic sequence, HBV has been classified into 8 different genotypes having distinct geographical distribution [15].

Genotype A can be regarded as pandemic but is most commonly found in Northern Europe, North America and Central Africa, while genotype B predominates in Asia (China, Indonesia and Vietnam). Genotype C is found in the Far East in Korea, China, Japan and Vietnam as well as the Pacific rim and Island Countries, while genotype D, which is also more or less pandemic, is found in the Mediterranean countries, the Middle East extending to India, North America and parts of the Asia-Pacific region. Genotype E is related to Africa while genotype F is found predominately in South America, including among Amerindian populations, and also Polynesia. Genotype G has been found in North America and Europe while the most recently identified genotype H has been reported from America [16].

Since the discovery of HBV subtypes, their impact on the natural course of infection has been studied mainly in the South-East Asia where HBV is hyper endemic with prevailing genotypes B and C. The clinical significance of different HBV genotypes has become increasingly recognized in patients with acute and chronic infection. The course of HBV infection depends on several factors such as host genetic factors, age and genetic variability of the virus [17,18]. Genotype C induces a more severe disease, has higher scores for fibrosis and is more prevalent in cirrhotic patients as compared to genotype B [19]. Seroconversion from HBeAg to anti-HBeAg positivity occurs much earlier in genotype B than genotype C carriers [20]. Genotype C is found to have lower HBV DNA level than genotype A, B and D in the HBeAg positive patients [21]. Taken together, these studies suggest well established pathogenic, epidemiological, clinical and therapeutic differences among HBV genotypes. However, the epidemiology of mixed HBV genotype infections is very less understood [22].

The epidemiological studies of various research groups indicated the predominant prevalence of genotype B and genotype C in South East Asian countries while the HBV/D is mainly found in Central Asia [23-25]. Apart from the initial studies reported mainly from Japan and China, it is now known that seven (A-G) HBV genotypes can be found in Asia. There are many studies focusing the prevalence of multiple genotype infections. In Pakistan, no such data is available up to now indicating the prevalence of HBV infected population with more than on genotype.

Most HBV infections result from sexual activity, injection-drug use, or occupational exposure [26]. Intravenous drug use and needle sick injuries have been identified as common modes of HBV transmission in the developing countries [27]. Therefore, the present study was conducted to assess the prevalent HBV genotypes in a well-known high risk group of Hepatitis B infection; Intra venous drug users (IDUs).

## Results

**Figure 1:**
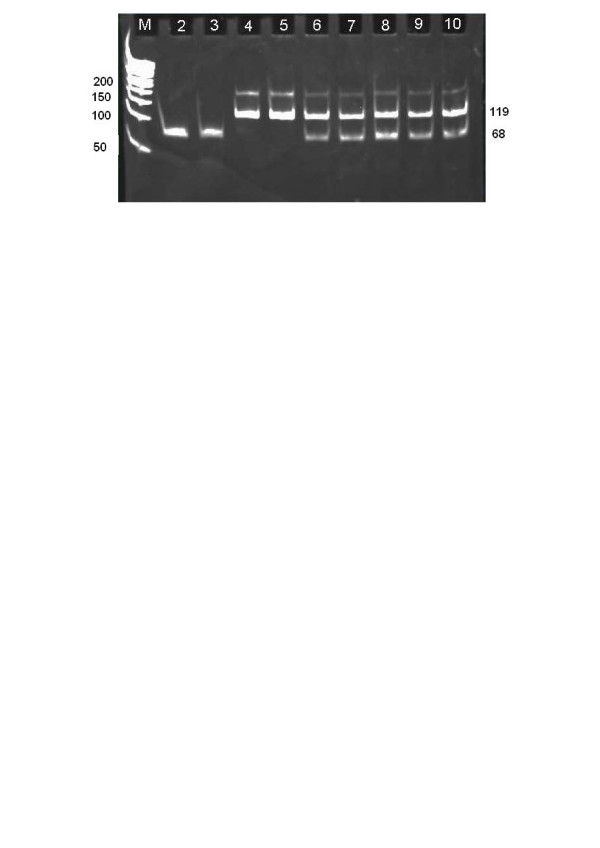
2.5% agarose gel showing genotype specific bands in patients infected with hepatitis B virus. M: 50 bp marker; Lane 2–3: HBV genotype A specific 68 bp band; Lane 4–5: HBV genotype D specific 119 bp band; Lane 6–10: genotype A and D specific 68 bp and 119 bp bands in patients with mixed genotype infections.

Out of the total 250 individuals, 56 (22.4%) subjects were found to be serological positive for HBsAg. All samples were further processed for genotyping. It was found that majority of the IDUs (35 out of 56) were infected with genotype D, making it the most frequently found genotype with prevalence rate of 62.5%. 15 (8.92%) individuals were found to be positive for genotype A while 16 (28.57%) drug addicts were found to be positive for genotype mixture A and D [Fig. 1].

## Discussion

In Pakistan, very little data is available about the IDUs reflecting an alarming situation of high infection rates among this highly vulnerable class of the society. Although most of these studies represent the data about HCV, for example, IDUs in Karachi have very high rates of hepatitis C (94%) which has also been documented in other reports as well [29,30]. In few studies, HBV status has been assessed like; 7.5% IDUs were found to be positive for Hepatitis B surface antigen in Karachi reported by the very recent joint study conducted by Enhanced HIV/AIDS Control Program, Government of Sindh Pakistan and United Nations Office for Drug Control and Crime Prevention (UNODC), 2007. According to a survey regarding prevalence of viral hepatitis among the high risk groups like frequent blood recipients and IDUs is 13.05% while 25.67% patients of Chronic Liver disease harbor Hepatitis B virus [31]. Inspection of 3 private health clinics in Hafizabad, Pakistan exposed disposable syringes and needles, used primarily for vitamin B complex, chloroquine, and penicillin, soaked in a bowl of tepid water. Extrapolation of study findings suggested 800 new cases of HCV and 109 HCV-related deaths in Hafizabad each year. Although this is referred to HCV but keeps equal importance as the risk of both HBV and HCV share equal importance using shared syringes. In another report, 350 IDUs were selected from a cohort study in Amsterdam of whom 70% injected recently, the prevalence of HIV, HBV and HCV were 31%, 68% and 65% respectively [32]. Also, awareness about hepatitis B and hepatitis C as a result of sharing needles and syringes is less (60%) [33].

According to the quantitative data on injection usage and unsafe injection practices, such as the reuse of unsterilized syringe or needles, obtained by reviewing the published articles and unpublished reports of the WHO, 18 studies presented convincing evidence on the association of unsafe injection practices and the transmission of blood borne viruses such as hepatitis B and C, Ebola, Lassa virus infections and malaria [34]. A simple mass-action model was applied to world census which showed that about 8–16 million HBV, 2.3–4.7 million HCV, and 80,000–160,000 HIV infections may result from unsafe injections each year [35].

Well established chains of transmission for blood-borne infections such as hepatitis and HIV exist in Pakistan. For example: needle sharing is common among the injecting drug users^36^. In 1999, UNDCP and UNAIDS Pakistan jointly studied the injecting drug users in Lahore, Pakistan's second largest city, finding an alarming high rate of hepatitis infection among them. Pakistan is a major transit and consumer country for opiates from neighboring Afghanistan, the world's largest producer of opium. As a result of the high levels of opium production in the region over the past two decades, Pakistan has now one of the highest addiction rates in the world [36]. According to the statistics provided by Anti-Narcotic Force, out of 4 million drug addicts in Pakistan, 3% are women, 12% of whom inject the drug which lead to high risk of Hepatitis B/C and HIV/AIDS.

Estimations of a local NGO working for the well-being of addiction free population in Pakistan reports a total of about 4.1 million drug addicts, of which 2 million are chronic heroin addicts. Since the early 1980s, political and economic changes within the region have facilitated a dramatic increase of poverty and social problems linked to the illegal production and marketing of opiates. Pakistan itself has succeeded in supply reduction; nevertheless, the NWFP is seriously affected. The main factors contributing to the problem of drug addiction are: high level of illiteracy and lack of social and life skills; daily easy availability of a deadly substance with analgesic and relaxing properties considering it a potential source of relief for all kinds of stress. In Pakistan, 53% of heroin addicts start experimenting with drugs at the age of 15–25 years [37]. NWFP has a striking figure of intravenous drug abusers locating in various regions of the province. Peshawar is located at the gateway of the transit trade route from Afghanistan easy availability of drugs at cheap prices are a permanent risk for Peshawar's youngster's and Pakistan's young population as a whole. Recent outbreak investigations of HBV in Sindh province, where the major etiological cause of the outbreak, was found to be the Intravenous drug injections among addicts signifies the IDUs as a major and potential transmission source of infection among the population.

In Pakistan, we have recently conducted a preliminary research project on prevalent genotypes of HBV in Pakistan (under publication) including general randomized population irrespective of major risk groups and have reported the prevalence of mixed genotypes infection in only 3% of total study subjects. However, here we have found that 28.57% IDUs are infected with multiple genotypes indicating very frequent horizontal transmission among the intravenous drug users.

The initial studies on HBV genotyping revealed that genotypes B and C are the most prevalent genotypes in Asian regions. It was because of the fact that all such studies were reported from Japan and China where genotype B and C are the most prevalent genotypes. Later on, it was found that all the seven HBV genotypes can be found in Asia [38]. For instance, the predominant genotypes in India are Genotype A and D [39]. The predominant HBV genotypes in Afghanistan were found to be genotype D [40]. Similarly, Zeng reported 1.6% patients infected with multiple HBV genotypes [41].

Mixed infection with more than one HBV genotypes is of growing interest from the epidemiological, virological, clinical and therapeutic points of view. Research studies on such multiple infections are the vital requirement to understand the events of recombination that has been reported between different HBV genotypes [42,43]. The multiple HBV genotypes determined in intravenous drug users (IDU) at Taiwan were as: mixed genotype A and B in 18 (5.5%); genotype B and C in 30 (9.2%); genotype B and D in 1 (0.3%); genotype A and C in 1 (0.3%); and mixed infections of genotype A, B, and C in 3 (0.9%) [44]. In Belgium, the HBV genotyping studies revealed multiple genotype infection rate of genotype A and genotype D to be 8% and 9% for blood donors and gastroenterology patients [45]. In Shenzhen (China), HBV infection rate with multiple genotypes was found to be 31% [46].

There are several studies reporting the existence of multiple HBV genotypes in various countries but mostly represent the figure related to patients irrespective of high risk groups like IDUs. For example, a study conducted on the patients from four regions of china reported that that mixed genotypes B and C was in 50.0% of the patients suggesting the mixed infection might lead to a severe damage of the liver tissue [47]. 26% of liver transplant patients at London were reported to harbor multiple genotypes of HBV [48]. In Tibet, the predominant HBV genotype is HBV C/D hybrid virus [49]. Hannoun *et al*. (2002) found 8% of HBV patients with genotype mixture [50]; Toan *et al*., (2006) found that chronic patients are more prone to be infected with more than one HBV genotype than acutely infected patients [38]; genotypes mixture in HBV patients is also common in Thailand [51]; 16% HBV cases were positive for HBV genotype mixture in France [52].

We have found 28.57% patients infected with multiple (more than one) HBV genotypes. It has already been documented that the HBsAg prevalence rate in pregnant women was 2.5% in Pakistan, out of which 17% and 61% were HBeAg and anti-HBeAg positive thus indicating the vertical transmission a less important cause of HBV transmission [53]. The present study reflects the importance of horizontal transmission as IDUs have several risky behaviors like sharing needles, cotton, syringes, multiple injections from a single drug source and jerking. The importance of such sources to identify remained the limitations of the study.

## Conclusion

This study brings basic information on the HBV positivity rate and genotype distribution among intravenous drug abusers of North West Frontier province of Pakistan. A possible proof of correlation with clinical and epidemiological characteristics will require further analysis. In conclusion, unsafe injections among drug addicts as well as medical practices occur routinely in Pakistan, implying a significant potential for the transmission of any blood borne pathogen. Unsafe injections currently account for a significant proportion of all new hepatitis B and C infections. This situation needs to be addressed immediately, as a political and policy issue, with responsibilities clearly defined at the country and community levels. Drug addicts should be at a prime importance as the targets of disease prevention and control programs in Pakistan as they do not stay permanently at a particular or specified area and are considered as mobile source of disease transmission.

## Methods

### Study Design

This study was completed during January 2005 to August 2006 at Serology laboratories, Department of Virology, National Institute of Health (NIH), Islamabad after receiving approval from the Research committee of NIH.

The study samples were collected from various locations of Peshawar, the capital of NWFP. Based on the facts that the intravenous drug abusers are highly vulnerable for blood borne infections and Peshawar is harboring a high number of drug addicts, the present study was designed to collect epidemiological information and molecular based analysis regarding hepatitis B virus, its current picture and disease status among the drug addicts living in North West frontier Province (NWFP) of Pakistan. 252 individuals were randomly selected. The study subjects were only males selected from all age groups ranging 12–65 years.

### Serological Testing

Blood sample was taken from all individuals after getting verbal informed consent. 8cc of venous blood was collected in a sterile vaccutainer and was referred to Serology laboratory, Department of Virology, NIH where centrifugation was done to separate sera. Sera were stored at -20°C until further processing. The individuals were screened for serological testing of HBV surface antigen (HBsAg) using AxSym HBsAg MEIA, Abbott Laboratories, IL, USA.

### Molecular analysis

Serological positive samples for HBsAg were further processed for HBV genotypes by the same methodology using genotype specific primers as described previously^28^. Briefly, DNA was extracted from 100 μl serum sample of 110 HBsAg positive patients using Biospin Blood Genomic DNA Mini-Prep Kit (Bioer Technology Co., Germany) according to the manufacturer's protocol, eluted in 70 μl buffer and stored at -20°C. Genotyping was performed using 50 μl reaction mixture was used containing 1X buffer, 20 μM primers, 2.4 μM dNTPs and 1.5U Taq polymerase. 40 cycles was performed at 95°C for 1 min, 50°C for 30 sec, 72°C for 30 sec. The amplified product was run on 2.5% agarose gel and visualized under U.V illuminator.

## Competing interests

The author(s) declare that they have no competing interests.

## Authors' contributions

SSZ and SAM designed the Research project and gave a critical view of manuscript writing, JAB and SS helped in collecting the samples. MMA and SS collected the epidemiological data and wrote the manuscript. AN analyzed the data statistically. SS and MA performed the serological and molecular assays. All the authors have read and approved the final manuscript.

## References

[B1] Zuckerman JN, Zuckerman AJ (2000). Current topics in hepatitis B. Journal of infections.

[B2] Lee WM (1997). Hepatitis B infection. New England Journal of Medicine.

[B3] Heerman KH, Gerlich WH, Michael C, Schaefer S, Thomssen R (1999). Quantitative detection of heopatitis B virus DNA in two international reference plasma preparartions. Journal of Clinical Microbiology.

[B4] Bowyer SM, Sim GM (2000). Relationship within and between the genotypes of Hepatitis B virus at point across the genome: footprints of recombination in certain isolates. Journal of General Virology.

[B5] Abe A, Kazuaki I, Take AT, Junko K, Nooki K, Satoshi T, Mkoto Y, Michinori K (2000). Quantification of Hepatitis B virus genomic DNA by Real-Time detection. Journal of Clinical Microbiology.

[B6] Mahoney FJ (1999). Update on diagnosis, management, and prevention of hepatitis B virus infection. Clinical Microbiology Reviews.

[B7] Yassin K, Awad R, Tebi Aj, Queder A, Laaser U (2002). Prevalence and risk factors of HBsAg in Gaza: implication for preventionand control. Journal of Infections.

[B8] Arboleda M, Castilho MC, Fonseca JC, Albuquerque BC, Saboia RC, Yoshida CF (1995). Epidemiological aspects of hepatitis B and D virus infection in the northern region of Amazonas, Brazil. Trans Roy Soc Trop Med Hyg.

[B9] Passos AD, Gomes UA, Figueiredo JF, do Nascimento MM, de Oliveira JM, Gaspar AM, Yoshida CF (1993). Influence of migration on the prevalence of serologic hepatitis B markers in a local rural community, analysis of prevalence by birth place. Revista De Saude Publica.

[B10] El-Sayed HF, Abaza SM, Mehanna S, Winch PJ (1997). The prevalence of hepatitis B and C infections among immigrants to a newly reclaimed area endemic for Schistosoma mansoni in Sinai, Egypt. Acta Tropica.

[B11] Chowdhury A, Santra A, Chaudhuri S, Ghosh A, Banerjee P, Mazumder DN (1999). Prevalence of hepatitis B infection in the general population: arural community based study. Trop Gastroenterolo.

[B12] Andre F (2000). Hepatitis B epidemiology in Asia: the Middle East and Africa. Vaccine.

[B13] (2000). HBV and HCV review article by JCPSP.

[B14] Orito E, Mizokami M, Ina Y, Moriyama EN, Kameshima N, Yamamoto M, Gojobori T (1989). Host dependent evolution and a genetic classification of the hepadnavirus family based on nucleotide sequence. Proceedings of National Academy of Sciences USA.

[B15] Okamoto H, Tsuda F, Sakugawa H, Sakugawa H, Sastrosoewignjo RI, Imai M, Miyakawa Y, Mayumi M (1988). Typing Hepatitis B virus by homology in nucleotide sequence: comparison of surface antigen subtypes. Journal of General Virology.

[B16] Kramvis A, Kew M, Francois G (2005). Hepatitis B virus genotypes. Vaccine.

[B17] Gunther S, Fischer L, Pult I, Sterneck M, Will H (1999). Naturally occurring variants of hepatitis B virus. Advances in Virus Research.

[B18] Hunt CM, McGill JM, Allen MI, Condreay LD (2000). Clinical relevance of hepatitis B viral mutations. Hepatology.

[B19] Kao JH, Chen PJ, Lai MY, Chen DS (2000). Hepatitis B genotypes correlate with clinical outcomes in patients with chronic hepatitis B. Gastroenterology.

[B20] Sumi H, Yokosuka O, Seki N (2003). Influence of Hepatitis B virus genotypes on the progression of chronic type B liver disease. Hepatology.

[B21] Westland C, Delaney W, Yang H (2003). Hepatitis B virus genotypes and virologic response in 694 patients in phase III studies of adefovir dipivoxil1. Gastroenterology.

[B22] Chen BF, Chen PJ, Jow GM, Sablon E, Liu CJ, Chen DS, Kao JH (2004). High prevalence of mixed genotype infections in hepatitis B virus infected intravenous drug users. J Med Virol.

[B23] Zhu B, Luo K, Hu Z (1999). Establishment of a method for classification of HBV genome and its application. Zhonghua Shiyan He Linchuang Bingduxue Zazhi.

[B24] Liu Cj, Kao Jh, Chen PJ, Lai My, Chen DS (2002). Molecular epidemiology of hepatitis B virus serotypes and genotypes in Taiwan. J of biomed Sciences.

[B25] Kato H, Ruzibakiev R, Yuldasheva N, Hegay T, Kurbanov F, Achundjanov B, Tuichiev L, Usuda S, Ueda R, Mizokami M (2002). Hepatitis B virus genotypes in Uzbekistan and validity of two different systems for genotyping. Journal of Medical virology.

[B26] Harpaz R, Von Seidlein L, Averhoff FM (1996). Transmission of hepatitis B virus to multiple patients from a surgeon without evidence of inadequate infection control. N Engl J Med.

[B27] Custer B, Sullivan SD, Hazlet TK, Iloeje U, Veenstra DL, Kowdley KV Global epidemiology of hepatitis B virus. Journal of clinical Gastroenterology.

[B28] Naito H, Hayashi S, Abe K (2001). Rapid and specific genotyping system for hepatitis B virus corresponding to six major genotypes by PCR using type specific primers. Journal of Clinical Microbiology.

[B29] United Nations Office on Drugs and Crime (2005). World Drug Report, Statistics, United Nations.

[B30] (2002). United Nations Drug Control Programme Global Assessment Programme on drug Abuse, Narcotics Control Division A-NFGoP: Drug Abuse in Pakistan. Results from the year 2000 National Assessment.

[B31] http://www.irin.org.

[B32] Van Ameijden EJ, Van den Hoek JA, Mientjes GH, Coutinho RA (1993). A longitudinal study on the incidence and transmission patterns of HIV, HBV and HCV infection among drug users in Amsterdam. Eur J Epidemiol.

[B33] Kuo I, Hasan S, Galai N, Thomas DL, Zafar T, Ahmed MA, Strathdee SA (2006). High HCV seroprevalnce and HIV drug use risk behaviours among injection drug users in Pakistan. Harem reduction Journal.

[B34] Simonsen L, Kane A, Lloyd J, Zaffran M, Kane M (1999). Unsafe injections in the developing world and transmission of bloodborne pathogens: a review. Bull World Health Organ.

[B35] Kane A, Lloyd J, Zaffran M, Simonsen L, Kane M (1999). Transmission of hepatitis B, hepatitis C and human immunodeficiency viruses through unsafe injections in the developing world: model-based regional estimates. Bull World Health Organ.

[B36] (2001). http://www.Unodc.org.

[B37] DOST Welfare Foundation, Pakistan. pak@dwf.pwr.sdnpk.undp.org.

[B38] Toan NL, Song LH, Kremsner PG, Duy DN, Binh VQ, Koeberlein B, Kaiser S, Kandolf R, Torresi J, Bock CT (2006). Impact of Hepatitis B virus genotypes and genotype mixtures on the course of liver disease in Vietnam. Hepatology.

[B39] Thakur V, Guptan RC, Kazim SN, Malhotra V, Sarin SK (2002). Profile, spectrum and significance of HBV genotypes in chronic liver disease patients in the Indian subcontinent. Journal of Gastroenterology and Hepatology.

[B40] Amini- Bavil-Olyaee S, Alavian SM, Adeli A, Sarrami-Forooshani R, Sabahi F (2006). Hepatitis B virus genotyping, core promoter and precore/core mutations among afghan patients infected with hepatitis B: a preliminary report. Journal of Medical Virology.

[B41] Zeng GB, Wen SJ, Wang ZH, Yan L, Sun J, Hou JL A novel hepatitis B virus genotyping system by using restriction fragment length polymorphism patterns of S gene amplicons. World J Gastroenterol.

[B42] Chen BF, Kao JH, Liu CJ, Chen DS, Chen PJ (2004). Genotypic dominance and novel recombinations in HBV genotype B and C co-infected intravenous drug users. J med Virol.

[B43] Kato H, orito E, Gish RG, Bzowel N, Newson M, Sugauchi F, Suzuki S, Ueda R, Miyakawa Y, Mizokami M (2002). Hepatitis B e antigen in sera from individuals infected with hepatitis b virus of genotype G. Hepatology.

[B44] Chen BF, Chen PJ, Jow GM, Sablon E, Liu CJ, Chen DS, Kao JH (2004). High prevalence of mixed genotype infections in hepatitis B virus infected intravenous drug users. J Med Virol.

[B45] Micalessi MI, De Cock L, Vranckx R (2005). Hepatitis B virus (HBV) genotyping in Belgian patients with chronic HBV infection. Clin Microbiol Infect.

[B46] Dai JY, Shi ZL, Dai Y, Du H, Chen DH, Wang SY (2004). Genotyping of HBV DNA in Shenzhen and clinical manifestations. Zhonghua Gan Zang Bing Za Zhi.

[B47] Zhu B, Luo K, Hu Z (1999). Establishment of a method for classification of HBV genome and it's application. Zhonghua Shi Yan He Lin Chuang Bing Du Xue Za Zhi.

[B48] Girlanda R, Mohsen AH, Smith H, Sablon E, Yuen MF, O'Grady J, Muiesan P, Rela M, Heaton N, Norris S (2004). Hepatitis B virus genotype A and D and clinical outcomes of liver transplantation for HBV-related disease. Liver Transpl.

[B49] Cui C, Shi J, Hui L, Zhuoma XX, Quni XX, Tsedan XX, Hu G (2002). The dominant hepatitis b virus genotype identified in Tibet is C/D hybrid. J Gen Virol.

[B50] Hannoun C, Krogsgaard K, Horal P, Lindh M (2002). Genotype mixtures of hepatitis B virus in patients treated with interferon. Journal of Infectious Diseases.

[B51] Jutavijittum P, Jiviriyawat Y, Yousukh A, Kunachiwa W, ToriYama K (2006). Genotypes of Hepatitis B virus among voluntary blood donors in northern Thailand. Hepatology Research.

[B52] Halfon P, Bourliere M, Pol S, Benhamou Y, Ouzan D (2006). Multicenter study of Hepatitis B virus genotypes in France: Correlation with liver fibrosis and hepatitis B e antigen status. Journa of Viral Hepatology.

[B53] Abbas Z, Jafri W, Shah SH, Khokhar N, Zuberi SJ (2004). Pakistan Society of Gastroenterology. PSG consensus statement on management of hepatitis B virus infection-2003. J Pak Med Assoc.

